# Real-World Evaluation of Microsatellite Instability Detection via Targeted NGS Panels in Routine Molecular Diagnostics

**DOI:** 10.3390/ijms26157138

**Published:** 2025-07-24

**Authors:** Petra Škerl, Vesna Vogrič, Vida Stegel, Vita Šetrajčič Dragoš, Olga Blatnik, Gašper Klančar, Srdjan Novaković

**Affiliations:** 1 Department of Molecular Diagnostics, Institute of Oncology Ljubljana, Zaloška 2, 1000 Ljubljana, Slovenia; pskerl@onko-i.si (P.Š.); vvogric@onko-i.si (V.V.); vstegel@onko-i.si (V.S.); vsetrajcic@onko-i.si (V.Š.D.); gklancar@onko-i.si (G.K.); 2 Faculty of Medicine, University of Ljubljana, Vrazov trg 2, 1000 Ljubljana, Slovenia; 3 Biotechnical Faculty, University of Ljubljana, Jamnikarjeva 101, 1000 Ljubljana, Slovenia; 4 Department of Pathology, Institute of Oncology Ljubljana, Zaloška 2, 1000 Ljubljana, Slovenia; oblatnik@onko-i.si

**Keywords:** microsatellite instability (MSI), biomarker, targeted next-generation sequencing (NGS), routine molecular diagnostics, tumor mutational burden (TMB), solid tumors

## Abstract

Microsatellite instability (MSI) is a clinically important biomarker for predicting responses to immune checkpoint inhibitors and identifying individuals with Lynch syndrome. Although MSI detection has been incorporated into Illumina’s next-generation tumor sequencing workflows, interpretation of the results remains challenging due to the absence of standardized thresholds and reporting criteria. In this retrospective study, we assessed the performance of MSI detection using Illumina’s targeted NGS panels—TruSight Tumor 170 and TruSight Oncology 500. The NGS-based MSI results were compared to those obtained by the reference method, MSI-PCR, across multiple tumor types in a real-world cohort of 331 cancer patients. The NGS method demonstrated high concordance overall (AUC = 0.922), though sensitivity was lower in colorectal cancers (AUC = 0.867) due to broader score variability and overlapping distributions. Our findings support the clinical utility of Illumina’s NGS-derived MSI scores for identifying MSI-H tumors, with a recommended MSI score cut-off value of ≥13.8%. Additionally, a borderline group was introduced, defined by an MSI score ranging from ≥8.7% to <13.8%. Within this range, the integration of TMB into the MSI classification workflow significantly improves diagnostic accuracy. For samples that remain inconclusive, orthogonal confirmation using MSI-PCR is advised to ensure accurate MSI classification.

## 1. Introduction

Microsatellite instability (MSI) is characterized by the accumulation of DNA variants, most commonly small deletions or insertions, in microsatellites due to defects in the DNA mismatch repair (MMR) genes. Microsatellites are short, repetitive DNA sequences distributed throughout the genome which are prone to errors during DNA replication. Normally, the MMR corrects these errors, but when it is impaired, mutations accumulate, leading to MSI [1,2,3]. A deficient MMR (dMMR) is usually caused by biallelic somatic or germline pathogenic variants in the MLH1, MSH2, MSH6, or PMS2 genes or by epigenetic changes like promoter hypermethylation of the MLH1 gene [1,3].

In recent years, MSI has become an important independent predictive biomarker for selecting patients with various solid tumors for treatment with immune checkpoint inhibitors [4,5,6]. Detecting MSI in tumors is also crucial to identifying Lynch syndrome patients. Consequently, patients with confirmed dMMR/MSI in their tumors should be referred for genetic counseling and testing for Lynch syndrome [2,7].

Currently, the most commonly accepted method for detecting dMMR in formalin-fixed, paraffin-embedded (FFPE) tumor tissue is immunohistochemical (IHC) staining for the expression of MLH1, PMS2, MSH2, and MSH6 proteins (MMR-IHC). For the assessment of MSI, the standard approach involves polymerase chain reaction (PCR)-based amplification of five to six mononucleotide or dinucleotide microsatellite loci, followed by fragment length analysis to identify instability [5,8]. Specifically, IHC detects the presence or absence of MMR proteins in tumor tissue and serves as a surrogate method for the detection of MSI. Loss of expression of one or more MMR proteins suggests a deficiency in the MMR genes, which is often associated with MSI. In contrast, PCR-based MSI testing evaluates the actual length variability in microsatellite regions of DNA, providing a direct molecular measurement of MSI status [5,8].

Both methods are widely available across various laboratories. However, MMR-IHC is the first test of choice and can provide insights into which MMR protein heterodimer is affected [5,6,8]. Nevertheless, it may also produce heterogeneous or ambiguous staining patterns, potentially leading to false-positive or false-negative interpretations [9,10]. Additionally, PCR-based MSI testing is usually used when there is disagreement or difficulty in interpreting IHC results [5,6,11]. However, it requires matched non-tumor tissue for accurate interpretation and typically assesses only a limited number of microsatellite loci.

An alternative approach for the molecular detection of MSI is next-generation sequencing (NGS) [5,6,8,12,13]. NGS offers a comprehensive method for detecting MSI by sequencing multiple microsatellite loci and simultaneously identifying clinically relevant genetic alterations, including mutations, gene amplifications, and tumor mutation burden (TMB), all within a single analysis. This integrative approach reduces the overall cost of tumor profiling and requires only a minimal amount of tissue, which is particularly advantageous in routine diagnostics. Moreover, a key advantage of NGS-based MSI detection is that it does not require matched non-tumor (normal) tissue as a reference, simplifying the testing process. In addition, NGS allows for comprehensive analysis across a broader range of microsatellite loci and is applicable to various tumor types beyond those traditionally associated with Lynch syndrome, making it a useful tool in both diagnostic settings [14].

In recent years, MSI detection has become an integrated component of broad multigene tumor profiling using commercially available targeted NGS panels such as Illumina’s TruSight Tumor 170 and TruSight Oncology 500, and also routinely used in our laboratory.

However, a major limitation of using targeted NGS for MSI detection in routine molecular diagnostics is the absence of standardized guidelines for test performance, result interpretation, and reporting. In contrast, PCR-based MSI testing benefits from established consensus recommendations regarding the selection of microsatellite (MS) loci panels and interpretation criteria [5,6]. Currently, no such guidelines exist for NGS-based MSI testing—neither in terms of the specific MS loci or panel size to be used nor the thresholds for defining MSI status. As a result, different studies have adopted varying definitions for the percentage of unstable MS loci required to classify a sample as microsatellite stable or microsatellite unstable, leading to considerable variability in MSI thresholds across NGS platforms and panels [10,15,16,17,18,19,20].

Therefore, in this retrospective study, we aimed to evaluate the analytical performance characteristics of NGS-based MSI detection using Illumina’s targeted sequencing panels, with PCR-based MSI results serving as the reference standard in a cohort of real-world patient samples. In addition, we aimed to establish the algorithm and workflow for MSI status interpretation to enable reliable MSI classification and the effective integration of NGS-derived MSI results into routine molecular diagnostic practice.

## 2. Results

### 2.1. Validation Cohort

The initial study cohort consisted of 331 tumor samples representing various cancer types. Of these, 321 tumor samples belonged within the spectrum of Lynch syndrome-associated malignancies (Table 1, Table S1).

**Figure 1:**
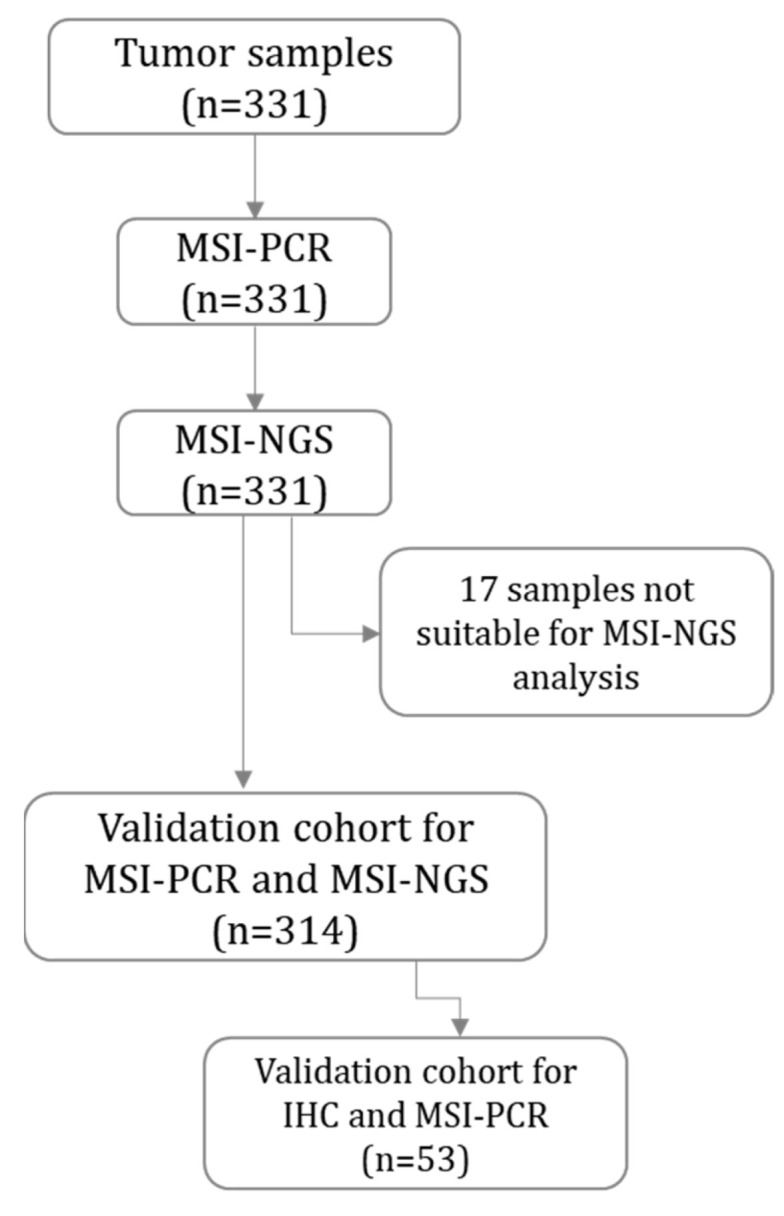
MSI-NGS validation cohort. The number of samples analyzed by NGS, PCR, and IHC for MSI/MMR status determination is shown within each box. MSI-PCR—MSI status was determined using PCR; MSI-NGS—microsatellite instability (MSI) status was determined using next generation sequencing (NGS); MMR-IHC—MMR status was determined using immunohistochemistry (IHC) staining of MMR proteins.

Among these, 17 tumors (5.1%) were assessed as unsuitable for NGS MSI analysis, as they did not meet the criteria of having at least 40 usable MSI sites evaluated during the NGS analysis. Consequently, the final validation cohort for MSI assessment by NGS included 314 tumor samples (Figure 1, Table 1, Table S1).

Among these, 28 tumors (8.92%) were classified as MSI-High (MSI-H) and 286 tumors (91.08%) were classified as MSI-Stable (MSI-S) based on fluorescent multiplex PCR analysis (MSI-PCR) targeting six mononucleotides repeat markers used as the reference (gold-standard) method. All MSI-H tumors harbored either a pathogenic variant in one of the mismatch repair (MMR) genes or MLH1 promoter hypermethylation (Table S2).

**Figure 2:**
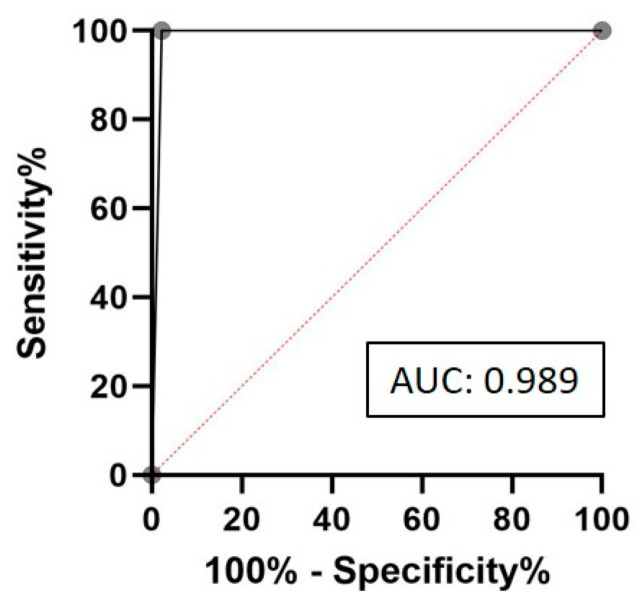
Receiver operating characteristic (ROC) curve analysis of MMR-IHC compared to MSI-PCR in tumor samples (*n* = 53).

Receiver operating characteristic (ROC) curve analysis was performed to assess the diagnostic performance of the widely used MMR-IHC method relative to the reference method, MSI-PCR, in a selected cohort of tumor samples (*n* = 53). This comparison is important because IHC, which infers MSI status based on MMR protein expression, may not directly reflect underlying microsatellite instability. ROC analysis enables a quantitative evaluation of IHC’s ability to correctly classify MSI status, identifying the optimal balance between sensitivity and specificity. As shown in Figure 2, the resulting ROC curve yielded an area under the curve (AUC) of 0.989, indicating excellent agreement between MMR-IHC and MSI-PCR and supporting the utility of IHC as a reliable screening tool in this context.

### 2.2. ROC Curve Analysis of MSI-NGS Diagnostic Performance

**Figure 3:**
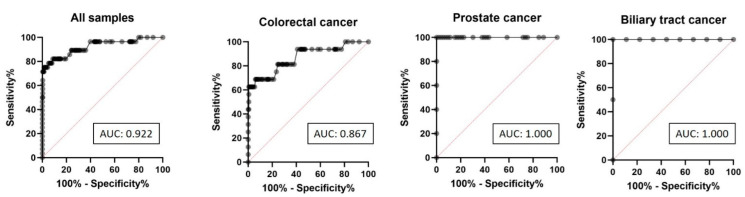
Receiver operating characteristic (ROC) curve analysis of MSI-NGS compared to MSI-PCR in all tumor samples (*n* = 314), colorectal cancer (*n* = 201), prostate cancer (*n* = 58), and biliary tract cancer (*n* = 11). AUC—area under the ROC curve value.

To evaluate the diagnostic performance of MSI detection using Illumina’s panels for targeted NGS (MSI-NGS), ROC curve analysis was conducted, using MSI-PCR as the reference method. In the overall validation cohort comprising 314 tumor samples, MSI-NGS demonstrated highly reliable discriminatory ability, yielding an area under the ROC curve (AUC) of 0.922, indicating high concordance with MSI-PCR (Figure 3).

Additional subgroup analyses by tumor type revealed variable AUC values. In colorectal cancer (*n* = 201), the AUC was 0.867, signifying lower, but still acceptable diagnostic accuracy of MSI-NGS in this subgroup (Figure 3). In prostate cancer (*n* = 58), an AUC of 1.00 was achieved, indicating perfect agreement between MSI-NGS and MSI-PCR. Similarly, in biliary tract cancer (*n* = 11), the AUC was 1.00, reflecting highly reliable performance; however, the small sample size in this subgroup should be considered when interpreting these results (Figure 3).

### 2.3. Determination of MSI Score Cut-Off Value and Evaluation of Diagnostic Performance Across Different MSI Score Cut-Offs

To determine the optimal MSI score threshold for classifying tumors as MSI-H using MSI-NGS, we evaluated the diagnostic performance of three candidate MSI score cut-off values based on the ROC curve: ≥5.7%, ≥8.7%, and ≥9.7%. These thresholds were selected based on analytical performance characteristics—including sensitivity, specificity, and overall accuracy—evaluated in our validation cohort (Table 2). For each cut-off, we examined key diagnostic metrics, including sensitivity, specificity, positive predictive value (PPV), negative predictive value (NPV), accuracy, and likelihood ratios (LRs), to determine the most robust and clinically relevant threshold (Table 2).

An MSI score cut-off of ≥5.7% yielded the highest sensitivity (78.5%), effectively identifying the largest proportion of true MSI-H cases. However, this threshold was associated with reduced specificity and a substantially lower PPV of 48.9%, indicating a high rate of false-positive results. A cut-off of ≥8.7% demonstrated a more balanced diagnostic profile, achieving high specificity (98.6%) and an improved PPV of 84.0%, while maintaining a modestly reduced sensitivity of 75.0% compared to the ≥5.7% threshold. The positive LR at this cut-off was 53, providing strong evidence to support true MSI-H classification following a positive test result. In contrast, the ≥9.7% cut-off had the highest specificity (99.7%) and PPV (95.2%), indicating extremely high confidence in MSI-H classification upon a positive result. However, this threshold exhibited the lowest sensitivity (71.4%), potentially missing nearly 30% of MSI-H cases within our validation cohort.

In addition, we defined the cut-off value based exclusively on true positive MSI-H samples. This threshold was determined at the point of 100% specificity, corresponding to a value of ≥13.8%. At this stringent cut-off, the positive predictive value (PPV) reached 100.0%, indicating complete confidence that all detected positive cases were true MSI-H cases. However, the sensitivity at this threshold was limited to only 50.0%, meaning that half of the true MSI-H cases were not detected.

To address this limitation and enhance the overall sensitivity of the MSI-NGS assay, a borderline group was introduced. This group encompasses samples with MSI scores ≥ 8.7% and < 13.8%, which are slightly below the high-specificity threshold, allowing for more nuanced interpretation and potential identification of additional true MSI-H cases. By incorporating the borderline category, we aim to reduce the number of false negatives and improve the clinical utility of the MSI-NGS assay, particularly in heterogeneous or low-tumor-content samples.

### 2.4. MSI Score Distributions in MSI-H and MSI-S Tumor Samples

**Figure 4:**
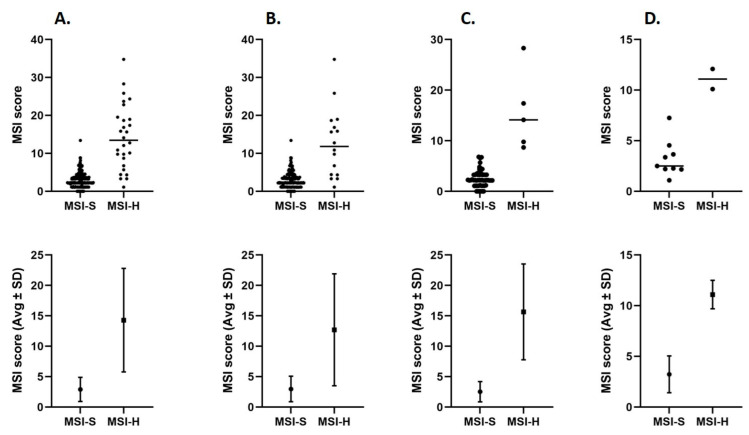
MSI score determined by NGS in MSI-S and MSI-H tumor samples. In the lower panel the mean MSI score (Avg) ± standard deviation (SD) is shown. MSI score (%) was determined using Illumina TruSight Oncology 500 or TruSight Tumor 170 kit. (**A**) All tumor samples (*n* = 314); (**B**) colorectal cancer (*n* = 201); (**C**) prostate cancer (*n* = 58); (**D**) biliary tract cancers (*n* = 11). MSI-S microsatellite stable tumor; MSI-H microsatellite high tumor.

MSI scores were determined for 314 tumor samples, comprising 28 MSI-H and 286 MSI-S cases (Table 3; Figure 4).

The mean MSI score for MSI-H tumors was 14.28% (SD ± 8.51), whereas MSI-S tumors had a mean score of 2.90% (SD ± 1.97). The 95% confidence interval (CI) for the MSI-H group ranged from 10.99 to 17.58, while for the MSI-S group, the CI ranged from 2.67 to 3.13 (Table 3). The *p*-value for the difference in means, calculated using Welch’s *t*-test, was <0.00001, indicating a statistically significant difference.

The range of MSI scores in the MSI-H group was 33.66%, spanning from 1.12% to 34.78%. In the MSI-S group, scores ranged from 0% to 13.41%, yielding a total range of 13.41% (Table 3). While there was some overlap at the lower end of the distributions, the upper limit of MSI-S scores (13.41%) remained below the maximum and below the mean MSI-H score.

In Figure 4 (Panel B), the MSI score distribution in colorectal cancer samples (*n* = 201) appears the most dispersed. While MSI-H cases still show higher average MSI scores than MSI-S cases, the separation between the two groups is less distinct than that observed in MSI-H prostate and biliary tract cancers (Panels C and D). Notably, MSI-H colorectal cancer cases exhibit a broader distribution of MSI scores, including individual cases with unexpectedly low values, resulting in partial overlap with the MSI-S group. The increased variability within the MSI-H cohort further diminishes the contrast between MSI-H and MSI-S tumors in colorectal cancer, making score-based classification in this subset more challenging, thus requiring greater caution.

**Figure 5:**
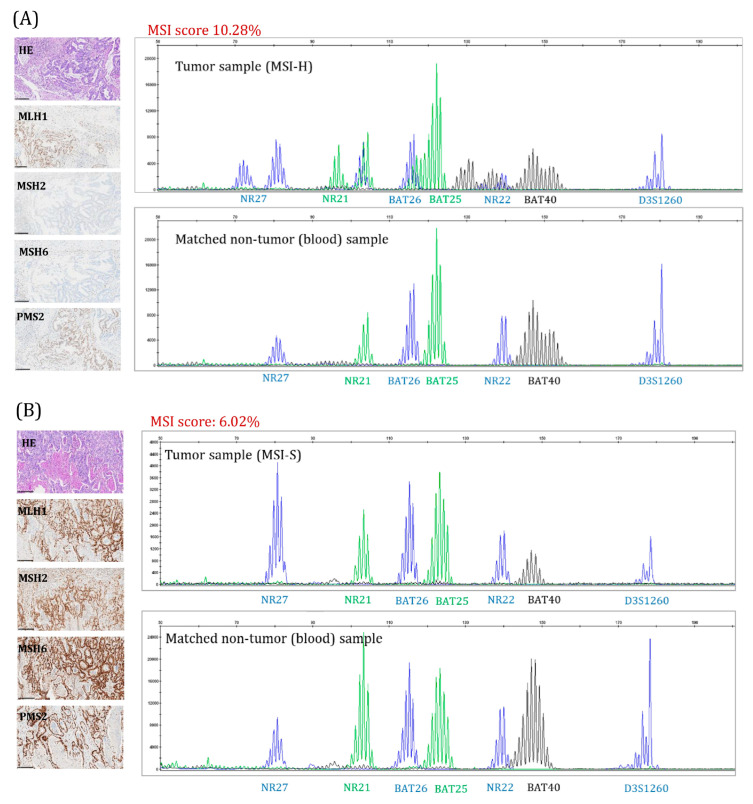
Microsatellite instability (MSI) status in representative colorectal tumor samples with corresponding MSI scores. dMMR/MSI was assessed using immunohistochemistry (IHC) staining of the mismatch repair (MMR) proteins—MLH1, MSH2, MSH6, and PMS2 (left panel)—and using fluorescent multiplex PCR, targeting six mononucleotides repeat markers (BAT25, BAT26, BAT40, NR21, NR22, and NR27), along with a polymorphic dinucleotide marker (D3S1260) as an internal control (right panel). (**A**) Sample 2058/21: MSI-High (MSI-H) tumor sample with determined MSI score of 10.28% using the NGS targeted panel; (**B**) Sample 2521/22: MSI-Stable (MSI-S) tumor sample with determined MSI score of 6.02% using the NGS targeted panel. HE—hematoxylin and eosin histological staining.

Figure 5 shows the results of MMR-IHC and MSI-PCR in representative MSI-H and MSI-S tumor samples, alongside their corresponding MSI scores.

### 2.5. Usable and Unstable Microsatellite Sites in MSI-H and MSI-S Tumor Samples

The average number of usable microsatellite instability (MSI) sites was comparable between MSI-S and MSI-H tumor samples. MSI-S tumors had a mean of 88.92 usable sites (SD ± 5.82), while MSI-H tumors had a slightly higher mean of 91.04 usable sites (SD ± 8.67) (Table 4). Across the entire cohort (*n* = 314), the overall mean number of usable sites was 89.11 (SD ± 6.15), indicating uniform assay performance and consistent microsatellite coverage irrespective of MSI classification (Table 4).

In contrast, a substantial difference was observed in the number of unstable MSI sites. MSI-H tumors had a mean of 13.18 unstable sites (SD ± 8.57), whereas MSI-S tumors had a mean of 2.56 unstable sites (SD ± 1.70) (Table 4). This difference was statistically significant (*p* < 0.00001), indicating a markedly higher level of microsatellite instability in MSI-H tumors compared to MSI-S tumors.

### 2.6. Association Between MSI Score, Tumor Cell Content, and Tumor Mutational Burden in MSI-H Tumor Samples

To investigate whether the proportion of tumor cells and tumor mutational burden (TMB) influence MSI score determination using MSI-NGS, MSI-H tumors (*n* = 28) were stratified based on an MSI score cut-off of ≥8.7%. Among MSI-H tumors with an MSI score ≥8.7 (*n* = 21), the mean MSI score was 17.67% (SD ± 6.80), and the average tumor cell content was 73.53% (SD ± 33.84). In contrast, MSI-H tumors with MSI scores <8.7% (*n* = 7) showed a substantially lower mean MSI score of 4.13% (SD ± 1.68) and a lower average tumor cell percentage of 56.43% (SD ± 24.31). Although the difference in mean tumor cell content between tumors with MSI scores ≥8.7% and tumors with MSI scores <8.7% did not reach statistical significance (*p* = 0.168), a trend toward higher tumor cellularity in samples with higher MSI scores was observed.

Similarly, the mean TMB was higher in the ≥8.7% group (38.15 mutations/Mb, SD ± 17.91) compared to the <8.7% group (27.14 mutations/Mb, SD ± 13.47), with a *p*-value of 0.109. While not statistically significant, the observed trend suggests that lower MSI scores may be associated with both reduced tumor cell content and a moderately decreased—but still elevated—TMB. Importantly, tumors with MSI scores <8.7% that were classified as MSI-H by PCR still exhibited high TMB values (mean = 27.14 mutations/Mb), indicating the presence of underlying genomic instability despite lower NGS-derived MSI scores (Table 5; Table S2).

### 2.7. Tumor Mutational Burden in MSI-H and MSI-S Tumor Samples

Tumor mutational burden (TMB) was significantly higher in MSI-H tumors (*n* = 28) compared to MSI-S tumors (*n* = 269) (Table 6).

The mean TMB in MSI-H tumors was 33.77 mutations/Mb (SD ± 18.75), while MSI-S tumors showed a mean of 6.90 mutations/Mb (SD ± 4.00). The 95% confidence intervals for the means were 25.46–42.08 for MSI-H and 6.40–7.40 for MSI-S, with no overlap between the groups. This difference was statistically significant (*p* < 0.000001).

### 2.8. Suggested Workflow for the Detection of MSI-H Tumors Based on MSI Scores and Tumor Mutational Burden (TMB)

**Figure 6:**
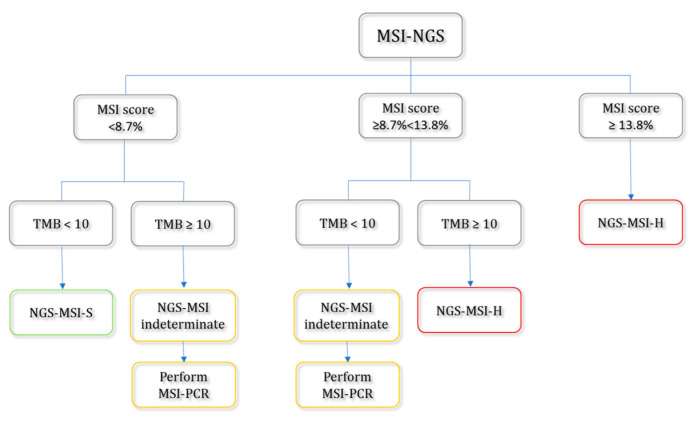
Diagnostic decision workflow for identifying MSI-H and MSI-S tumors using NGS-derived MSI scores and TMB.

Based on data from our validation cohort, we propose a diagnostic workflow that integrates MSI scores with TMB to enhance the identification of MSI-H tumors (Figure 6).

According to the proposed workflow, tumor samples are categorized into three groups based on the MSI score and TMB thresholds: MSI-Stable (NGS-MSI-S; *n* = 191))—tumors with an MSI score < 8.7% and TMB < 10 mutations/Mb; MSI-High (NGS-MSI-H; *n* = 23)—tumors are classified as MSI-H if the MSI score is ≥13.8%, or if the MSI score is ≥8.7% and the TMB is ≥10 mutations/Mb; and the NGS-MSI-indeterminate group (*n* = 85), where further MSI assessment by MSI-PCR is recommended. The indeterminate group includes tumors that do not clearly fall into the NGS-MSI-S or NGS-MSI-H categories—specifically, those with an MSI score between 8.7% and 13.8% and TMB < 10 or those with an MSI score < 8.7% and TMB ≥ 10 are recommended for further assessment by MSI-PCR for definitive MSI status determination.

Integration of TMB into the diagnostic workflow markedly improved overall diagnostic performance. When applying an MSI score threshold of ≥8.7% alone for classifying tumors as NGS-MSI-H, the diagnostic accuracy reached 96.5%. However, the incorporation of TMB into the classification criteria, as shown in Figure 6, further increased accuracy to 99.6% in the combined NGS–MSI-S and NGS–MSI-H patient subgroups (*n* = 214), as previously described. In this subgroup, the PPV and NPV were 100.0% and 99.4%, respectively.

## 3. Discussion

In recent years, high microsatellite instability (MSI-H) has emerged as a key predictive biomarker for identifying patients with various solid tumors who may benefit from immune checkpoint inhibitor therapy [4,5,6]. Consequently, the rigorous validation of MSI detection methods is essential to ensure accurate patient selection for immunotherapy. In addition, MSI plays a crucial role in identifying individuals with Lynch syndrome [2,7].

While MSI detection has become an integral component of comprehensive multigene tumor profiling—commonly performed using commercially available comprehensive targeted NGS panels such as Illumina’s TruSight Tumor 170 and TruSight Oncology 500—there remain notable limitations in clinical interpretation. Although these platforms provide detailed outputs, including using MSI scores to infer MSI status, the results are often presented without standardized interpretation guidelines. This lack of interpretive context can hinder clinical decision-making, particularly in borderline or ambiguous cases, and underscores the need for clear thresholds, harmonized reporting criteria, and proper clinical annotation to ensure actionable insights. Moreover, utilizing MSI results obtained through comprehensive targeted NGS panels offers several practical advantages. This approach is both time- and cost-efficient, as it allows the simultaneous analysis of multiple biomarkers—including MSI status—in a single assay. Importantly, it eliminates the need for matched normal (non-tumor) tissue, which can be difficult to obtain in routine clinical settings. These benefits make NGS-based MSI assessment highly suitable for integration into standard diagnostic workflows, streamlining precision oncology efforts and supporting timely therapeutic decision-making.

Despite the growing adoption of comprehensive targeted NGS panels in clinical oncology, there remains a limited number of studies specifically evaluating their performance in determining MSI status [10,12,15,16,17,20,21].

In this study, we evaluated the performance of an Illumina’s targeted NGS panel-based approach for MSI detection by comparing MSI scores, tumor mutational burden (TMB), and tumor cell content across MSI-H and MSI-S tumors, and benchmarking results against established methods such as MSI-PCR and MMR-IHC. Our findings support the clinical utility of MSI scoring derived from Illumina’s NGS panels TruSight Tumor 170 and TruSight Oncology 500, while also highlighting key considerations for improving sensitivity and diagnostic accuracy.

We first assessed the diagnostic performance of MSI scoring by targeted NGS panels (MSI-NGS), using MSI-PCR as the benchmark. The overall AUC for MSI-NGS was 0.922, indicating high concordance and strong diagnostic accuracy. However, subgroup analyses revealed tumor-type-specific variability. While prostate and biliary tract cancers achieved perfect MSI classification (AUC = 1.00), colorectal cancers exhibited a lower AUC of 0.867, likely reflecting greater biological heterogeneity and a broader MSI score distribution in this group. These findings are consistent with several other published studies, which have reported good concordance between NGS-based MSI assessment and reference methods such as MSI-PCR or MMR-IHC, particularly when using well-validated pipelines and optimized thresholds [10,12,13,16,17,19,22,23]. However, discrepancies in colorectal cancer NGS-based MSI classification have also been noted by others, further emphasizing the importance of tumor-specific validation and the integration of additional genomic markers such as tumor mutational burden (TMB) to support clinical interpretation [10,16,17].

To optimize diagnostic accuracy, three NGS-based MSI score cut-off values (≥5.7%, ≥8.7%, and ≥9.7%) for the classification of tumors as NGS–MSI-H were evaluated for diagnostic performance. A threshold of ≥8.7% offered the best balance of sensitivity (75.0%) and specificity (98.6%), with a positive predictive value of 84.0%, making it the most clinically appropriate cut-off for routine diagnostics. While the ≥9.7% threshold provided the highest specificity (99.7%) and PPV (95.2%), it resulted in reduced sensitivity (71.4%) and risked missing nearly 30% of true MSI-H cases. Furthermore, a cut-off value of ≥13.8% was defined based solely on confirmed MSI-H cases at the point of 100% specificity, achieving a PPV of 100.0% and ensuring all identified MSI-H samples were true positives, although this stringent threshold reduced sensitivity to 50.0%, missing half of the true MSI-H cases.

In other studies, using Illumina’s TruSight Oncology 500, including recent recommendations from Illumina, a higher MSI score threshold—such as ≥20.0%—has been proposed to classify tumors as MSI-H [16,20,21]. However, applying this stringent cut-off to our patient cohort would have resulted in a significant underdiagnosis of MSI-H cases. Specifically, only 6 out of 28 MSI-H tumor samples (21.4%) in our dataset exceeded the 20.0% threshold, meaning that nearly 80% of MSI-H cases would have been missed. This finding highlights the importance of validating MSI thresholds using diverse, real-world patient samples and suggests that more moderate cut-offs may be required to preserve diagnostic sensitivity across varied clinical populations.

Although we applied a relatively low MSI score cut-off compared to other published studies, the sensitivity of targeted NGS for detecting MSI-H tumors in our cohort was lower (at 75%). In contrast, other studies have reported sensitivities exceeding 90% when using similar NGS-based approaches [16,20,21]. This discrepancy may be attributed to differences in tumor types, tumor cell content, sample quality, or sequencing depth. It also highlights the need for careful optimization of cut-off values and validation across diverse clinical settings to ensure robust performance and minimize the risk of false-negative results in real-world patient tumor samples.

On the other hand, MSI scores demonstrated clear separation between MSI-H and MSI-S tumors, with MSI-H samples exhibiting significantly higher mean scores (14.28% ± 8.51) compared to MSI-S samples (2.90% ± 1.97, *p* < 0.00001) (Table 3). However, variability within the MSI-H group was notable, particularly in colorectal cancers, where MSI-H samples showed a broader range and included cases with low MSI scores (Figure 4). This intra-group heterogeneity diminishes the discriminatory power of a fixed MSI score cut-off and underscores the importance of contextual interpretation, particularly in tumor types with broad MSI score distributions. To address this, it was reasonable to implement a “borderline MSI” category—defined by an intermediate MSI score range, such as between 8.7% and 13.8%—to capture cases that fall near the threshold of MSI classification (Figure 6). Introducing this borderline category allows for a more refined approach to MSI interpretation and helps to mitigate the risk of false-negative or ambiguous results. In such cases, the use of complementary methods, such as MSI-PCR, is strongly recommended for confirmatory analysis [16,17]. This strategy enhances diagnostic confidence, particularly when treatment decisions or hereditary cancer syndromes like Lynch syndrome are being considered. Similar approaches have been adopted in other studies, where an “inconclusive” or “borderline” MSI group has been defined to accommodate analytical and biological variability, further supporting the value of a tiered classification system in real-world diagnostics [16,17].

To further explore the cause of unexpectedly low MSI scores in certain colorectal cancer samples, we investigated whether tumor cell content could be a contributing factor. Specifically, we compared the tumor cell percentage in MSI-H samples with MSI scores <8.7% to those with scores ≥8.7%. The analysis revealed that MSI-H tumors with lower MSI scores had a reduced average tumor cell content (56.43 ± 24.31%) compared to those with higher scores (73.53 ± 33.84%), although this difference did not reach statistical significance (*p* = 0.168). These findings suggest that reduced tumor cellularity may lead to the underestimation of MSI scores, potentially resulting in false-negative classifications, especially for cases near the threshold. This observation was also reported by Al-Kateb et al., who demonstrated that MSI classification was discordant when tumor content was ≤20%, with a 0% positive predictive agreement [20].

In contrast, tumor mutational burden (TMB) was significantly higher in all MSI-H tumors compared to MSI-S tumors (33.77 ± 18.75 vs. 6.90 ± 4.00 mutations/Mb; *p* < 0.000001), reinforcing the well-established association between high MSI and a hypermutated phenotype (high TMB) [3,5,13,24]. Notably, even within the MSI-H group, tumors with MSI scores <8.7% maintained elevated TMB levels (27.14 ± 13.47 mutations/Mb), indicating that TMB may serve as a complementary marker of genomic instability. This suggests that TMB assessment could aid in identifying MSI-H tumors with borderline MSI scores and improve the accuracy of MSI classification in such ambiguous cases (Figure 6).

By integrating TMB into the NGS-MSI classification workflow, diagnostic performance improved markedly. The incorporation of TMB and the introduction of the ‘NGS–MSI-indeterminate’ group, as outlined in the proposed interpretation workflow (Figure 6), increased the overall diagnostic accuracy to 99.6% in the combined NGS–MSI-S and NGS–MSI-H patient subgroups. Using this workflow, the PPV and NPV in the combined subgroup also improved significantly—to 100.0% and 99.4%, respectively—compared to 84.0% and 97.6% when using the MSI score alone as the classification criterion. These findings underscore the superior reliability of the combined approach for accurately identifying MSI-H tumors. A similar approach was employed by Kang et al., who also introduced a borderline MSI category and demonstrated that the incorporation of TMB significantly enhanced MSI classification accuracy. By combining MSI score interpretation with TMB data, they improved the sensitivity of MSI detection to 100%, while achieving a positive predictive value (PPV) of 97.87% [16]. These findings underscore the utility of integrating multiple genomic biomarkers to resolve borderline or ambiguous MSI cases and reinforce the value of a multi-parameter strategy in optimizing diagnostic precision.

An additional advantage of using a comprehensive targeted NGS panel, compared to PCR or IHC, is the ability to assess multiple relevant biomarkers in a single assay. Alongside MSI and TMB status, these panels can simultaneously detect pathogenic variants in MMR genes—including *MLH1*, *MSH2*, *MSH6*, and *PMS2*—as well as the presence of the *BRAF* V600E mutation. This integrated approach provides important insights for the differential diagnosis of sporadic versus hereditary MSI-H tumors and supports the clinical work-up for Lynch syndrome screening. In our patient cohort, 19 of 28 MSI-H tumors harbored a pathogenic variant in one or more MMR genes (Table S2). Additionally, five cases exhibited both MLH1 promoter hypermethylation and a *BRAF* V600E mutation—findings typically associated with sporadic MSI-H colorectal cancers. Four other MSI-H tumors showed only MLH1 promoter hypermethylation. These findings highlight the utility of comprehensive NGS panels in providing a molecular context that can guide further germline testing and inform personalized patient management strategies.

It is important to note that not all tumor samples from real-world cancer patients are suitable for NGS-based MSI assessment. In our patient cohort, 5.1% of samples were estimated as unsuitable for MSI scoring via NGS, as they failed to meet the minimum requirement of evaluating at least 40 usable MSI loci during analysis. This limitation can be attributed to factors such as low DNA quality, poor coverage, or inadequate tumor content. For such cases, alternative methods like MSI-PCR or MMR-IHC remain more reliable and feasible options, ensuring that MSI status can still be accurately determined when NGS is not applicable.

Although our study is based on a relatively limited sample size, it provides valuable real-world evidence on the performance of targeted NGS panels for MSI detection. In light of the current scarcity of comprehensive validation studies in this field—particularly across diverse tumor types and patient populations—our findings contribute to the growing body of evidence supporting the use of NGS-based MSI assessment in clinical practice [5,6,10,16,17,20]. However, several limitations must be acknowledged. The most significant limitation is the small number of MSI-H tumors included in the cohort, which may have influenced the accuracy and predictive performance metrics, particularly sensitivity and positive predictive value. As MSI-H tumors are typically underrepresented in many real-world datasets, larger studies are necessary to validate these findings and ensure robust statistical power. Additionally, the majority of samples in our analysis were processed using the Illumina TruSight Tumor 170 panel, which has a more limited genomic footprint compared to larger panels such as the TruSight Oncology 500. This smaller panel size could potentially restrict the detection of certain MSI-associated variants or impact TMB estimation. Future studies should aim to expand the analysis to include larger and more diverse cohorts, incorporate multiple NGS platforms, and assess the performance across a wider range of tumor types. Such efforts will be critical to further establishing the clinical utility, reproducibility, and reliability of NGS-based MSI detection in routine oncology workflows.

In summary, our findings support the clinical utility of NGS-derived MSI scores obtained from Illumina’s targeted panels for the identification of MSI-H tumors, consistent with previous reports [16,20]. As also demonstrated by other investigators, the integration of TMB can further enhance diagnostic accuracy, particularly in borderline cases [16]. The added value of our study lies in the development and proposal of a practical diagnostic workflow for NGS-MSI results interpretation that incorporates both MSI score and TMB for use in real-world diagnostic settings. This proposed interpretation workflow introduces a lower MSI score threshold (≥8.7%) than previously described. The inclusion of TMB alongside MSI scoring refines classification accuracy, reduces false-negative rates, and enhances confidence in identifying MSI-H tumors, particularly in ambiguous or low-score cases. The workflow also introduces the NGS-MSI-indeterminate group, which includes cases where classification remains uncertain—particularly when MSI scores fall within the borderline range—and we recommend that orthogonal confirmation using MSI-PCR to ensure diagnostic confidence be performed. Importantly, the proposed workflow is adaptable across tumor types and it offers a structured, evidence-based approach to guide precision oncology decision-making. By bridging the gap between molecular profiling and clinical application, this workflow has the potential to improve the selection of patients for immunotherapy in routine molecular diagnostic.

## 4. Materials and Methods

### 4.1. Patients and Tumor Samples

Our study group included 331 patients diagnosed with different cancer types (Table 1) treated at the Institute of Oncology Ljubljana between 2019 and 2022. All patients provided written informed consent. The study was retrospective and was approved by the Institutional Review Board of the Institute of Oncology Ljubljana and Ethics Committee of the Institute of Oncology Ljubljana (Permission No. ERIDPRO-0026/2024).

Tumor samples were formalin-fixed and paraffin-embedded (FFPE) and were evaluated by a dedicated pathologist according to the established laboratory protocol. Sample evaluation comprised histological examination and conformation of the tumor type, determination of the percentage of tumor cells in the sample, and selection of the representative tumor block for DNA isolation. For each tumor sample, a matching control sample (normal, non-tumor tissue or blood sample) was obtained.

### 4.2. DNA Isolation

Genomic DNA from tumor and control non-tumor tissue FFPE samples was isolated using the QIAamp DNA FFPE Tissue kit (Qiagen GmbH, Hilden, Germany) or the MagMax DNA/RNA kit (ThermoFisher Scientific, Woodward, St. Austin, TX, USA) according to the established laboratory protocol and previously described [25,26]. DNA from control blood samples was extracted using the QIAamp DNA Blood Mini Kit (Qiagen) according to the manufacturer’s protocol. Extracted DNA was quantified using the Qubit dsDNA HS Assay kit and the fluorimeter Qubit 3.0. (both ThermoFisher Scientific, Eugene, OR, USA).

### 4.3. MSI Detection Using PCR (MSI-PCR)

Microsatellite instability (MSI) was assessed using a validated in-house fluorescent multiplex PCR assay targeting six mononucleotides repeat markers, BAT25, BAT26, BAT40, NR21, NR22, and NR27, which has been described previously [26,27]. Testing was performed on DNA extracted from tumor tissue and matched normal (germline) control samples. In addition to the mononucleotide markers, a highly polymorphic dinucleotide marker, D3S1260, was included as an internal control to confirm identity matching between tumor and normal samples. A microsatellite marker was considered unstable if the fragment length differed by two or more base pairs between the tumor and the matched germline DNA. Based on the number of unstable markers, MSI status was classified as follows: MSI-High (MSI-H)—instability present in three or more of the six mononucleotide markers; MSI-Low (MSI-L)—instability observed in one or two markers; and microsatellite stable (MSI-S)—no instability detected in any of the markers. For the purposes of this study, tumors with instability in two or fewer markers were categorized as MSI-S, including samples that would otherwise be classified as MSI-L.

### 4.4. MSI Detection Using Next Generation Sequencing (MSI-NGS) and MSI Score Determination

Next-generation sequencing (NGS) of tumor DNA was performed using the NextSeq 550 Sequencing System (Illumina, San Diego, CA, USA). Library preparation was conducted using either the TruSight Tumor 170 DNA kit (*n* = 305; Table S1) or the TruSight Oncology 500 kit (*n* = 26; Table S1), both from Illumina, as previously described by our group [26,28].

Bioinformatic analysis was conducted using TruSight Tumor 170 BaceSpace App and TruSight Oncology 500 Local App (Illumina) pipelines, as previously reported [26]. Microsatellite instability (MSI) status was evaluated using a tumor-only algorithm that interrogates 130 non-coding homopolymer regions (MSI loci) common to both NGS panels. The MSI score was defined as the percentage of unstable MSI loci among all usable loci in each sample (MSI score (%) = no. of unstable MSI sites/total assessed usable MSI sites). According to the manufacturer’s recommendations, a sample was considered suitable for MSI scoring if at least 40 usable MSI loci were available for analysis following bioinformatic processing (Illumina).

### 4.5. Tumor Mutational Burden (TMB)

Tumor mutational burden (TMB) was defined by counting the number of somatic non-synonymous and synonymous base substitution and small indels (insertions and deletions less than 20 nucleotides), with a VAF of ≥5%, in the coding regions per megabase (mut/Mb). The cut-off value for TMB-high was set at 10 mut/Mb, in accordance with the KEYNOTE-158 trial, which supported FDA approval of pembrolizumab for the treatment of solid tumors exhibiting high tumor mutational burden [29].

### 4.6. Immunohistochemical (IHC) Analysis

Immunohistochemical (IHC) analysis of MMR protein expression (MMR-IHC) was performed using a standard procedure by dedicated pathologist. Briefly, IHC staining was performed on 2 to 4 µm FFPE tissue sections using fully automated IHC system Ventana BenchMark Ultra (Ventana Roche, Tucson, AZ, USA). For this purpose, commercially available monoclonal antibodies for MLH1 (clone ES05, #M3640; diluted 1:20) (DAKO Agilent, Santa Clara, CA, USA), PMS2 (clone EP51, #M3647; diluted 1:100) (DAKO Agilent), MSH2 (clone G219-1129, #760-5093; RTU) (Ventana Roche), and MSH6 (clone SP93, #760-5092; RTU) (Ventana Roche) were used. Primary antibodies were visualized using a three-step multimer detection system OptiView DAB IHC Detection Kit (Ventana Roche) following the manufacturer’s protocol. MLH1 and PMS2 detection was enhanced using OptiVew Amplification Kit (Ventana Roche). All protocols passed external quality assessment in both NEQAS and NORDIQC. Normal appendix and colon carcinomas with confirmed protein expression or loss of protein expression were used as controls.

### 4.7. Methylation-Specific MLPA (MS-MLPA)

Methylation status of the promoter region of the MLH1 was determined using methylation-specific multiplex ligation dependent probe amplification (MS-MLPA). Probemix ME011 Mismatch Repair Genes was used according to the manufacturer’s instruction (MRC Holland, Amsterdam, The Netherlands). For validation purposes, the methylation status was performed in MSI-H tumor samples without a pathogenic variant in MMR genes.

### 4.8. Statistical Analysis

All statistical analyses (receiver operating characteristic (ROC) curve analysis, area under the ROC curve value (AUC), specificity, sensitivity, positive predictive value, negative predictive value, accuracy, likelihood ratio, mean MSI score, and standard deviation) were calculated using GraphPad Prism version 9.5.1. for Windows (GraphPad Software, Boston, MA, USA, www.graphpad.com). The positive predictive value (PPV) was defined as the number of true positives/number of positive calls (true positives or MSI-H were determined by MSI-PCR). The negative predictive value was defined as number of true negatives/number of negatives calls (true negatives or MSI-S were determined by MSI-PCR). Likelihood ratio was defined as the ratio of the true positive rate to the false positive rate. Statistically significance was determined using Welch’s *t*-test.

## Figures and Tables

**Table 1:** Summary of tumor types and number of samples in the initial testing group and validation cohort.

Tumor Type	Lynch Syndrome-Associated Cancer	Initial Tumor Samples Group (*n*)	Validation Cohort (*n*)
Colorectal cancer	yes	209	201
Prostate cancer	yes	64	58
Gastric cancer	yes	16	16
Biliary tract cancers	yes	12	11
Pancreatic cancer	yes	13	12
Endometrial cancer	yes	4	3
Urothelial cancer	yes	3	3
Esophageal cancer	no	3	3
Origo ignota	no	2	2
Malignant melanoma	no	1	1
Bone cancer	no	1	1
Penile cancer	no	1	1
Adrenal gland tumor	no	1	1
Breast cancer	no	1	1
All tumor samples		331	314

**Table 2:** Analytical validation of MSI-NGS for the detection of microsatellite instability (MSI) compared to MSI-PCR. Analytical parameters are shown for different cut-off values of MSI scores determined by MSI-NGS in the validation cohort of tumor samples (*n* = 314).

Analytical Parameter	MSI Score Cut-Off Value (%)			
	≥5.7	≥8.7	≥9.7	≥13.8
Specificity % (95% CI)	91.9 (88.2–94.6)	98.6 (96.5–99.5)	99.7 (98.1–100.0)	100.0 (98.7–100.0)
Sensitivity % (95% CI)	78.5 (60.4–90.0)	75.0 (56.6–87.3)	71.4 (52.9–84.8)	50.0 (32.6–67.4)
Positive predictive value %	48.9	84.0	95.2	100.0
Negative predictive value %	97.8	97.6	97.2	95.3
Accuracy	90.8	96.5	97.2	95.5
TPR + TNR	170.5	173.6	171.1	150.0
Likelihood Ratio	10	53	204	/

TPR, true positive rate or sensitivity; TNR, true negative rate or specificity; TPR + TNR, sensitivity + specificity).

**Table 3:** Descriptive statistics of MSI scores in MSI-H and MSI-S tumor samples.

MSI Score (%)	MSI-H (*n* = 28)	MSI-S (*n* = 286)
Mean ± SD	14.28 ± 8.51	2.90 ± 1.97
Minimum	1.12	0
Maximum	34.78	13.41
Range	33.66	13.41
Lower 95% CI of mean	10.99	2.67
Upper 95% CI of mean	17.58	3.13

Microsatellite stable tumors (MSI-S) and microsatellite high tumors (MSI-H) were determined using MSI-PCR.

**Table 4:** Usable MSI sites determined in the validation cohort of tumor samples (*n* = 314).

MSI Status	MSI-S	MSI-H	All Samples
No. of usable MSI sites (mean ± SD)	88.92 ± 5.82	91.04 ± 8.67	89.11 ± 6.15
No. of unstable MSI sites (mean ± SD)	2.56 ± 1.70	13.18 ± 8.57	

Microsatellite stable tumors (MSI-S) and microsatellite high tumors (MSI-H) were determined using MSI-PCR. The number of usable MSI sites and number of unstable MSI sites were determined using targeted NGS panel sequencing.

**Table 5:** Comparison of MSI scores with tumor cell percentages and TMB based on MSI score cut-off value in MSI-H tumors (*n* = 28).

MSI Score Cut-Off Value in MSI-H Tumor Samples	MSI Score Cut-Off Value ≥8.7% (*n* = 21)	MSI Score Cut-Off Value <8.7% (*n* = 7)
MSI score (%) (mean ± SD)	17.67 ± 6.80	4.13 ± 1.68
% of tumor cells in tumor sample (mean ± SD)	73.53 ± 33.84	56.43 ± 24.31
TMB (mean ± SD)	38.15 ± 17.91	27.14 ± 13.47

Microsatellite high tumors (MSI-H) were determined using MSI-PCR (gold-standard method). TMB—tumor mutational burden.

**Table 6:** Tumor mutational burden (TMB) in MSI-H and MSI-S tumor samples.

TMB	MSI-H (*n* = 28)	MSI-S * (*n* = 269)
Mean ± SD	33.77 ± 18.75	6.90 ± 4.00
Minimum	4	0
Maximum	82	22
Range	78	22
Lower 95% CI of mean	25.46	6.4
Upper 95% CI of mean	42.08	7.4

Microsatellite stable tumors (MSI-S) and microsatellite high tumors (MSI-H) were determined using MSI-PCR. * Two MSI-S tumor samples with a POLE pathogenic variant were excluded from calculations; in 15 tumor samples, TMB was not determined.

## Data Availability

The raw data supporting the conclusions of this article will be made available by the authors on request.

## Supplementary Materials

The following supporting information can be downloaded at: https://www.mdpi.com/article/10.3390/ijms26157138/s1.

## Abbreviations

The following abbreviations are used in this manuscript:

MSI	Microsatellite instability
MSI-H	Microsatellite instability high
MSI-S	Microsatellite stable
MS loci	Microsatellite loci
IHC	Immunohistochemistry
PCR	Polymerase chain reaction
NGS	Next-generation sequencing
MMR	Mismatch repair
TMB	Tumor mutational burden
ROC	Receiver operating characteristic curve
AUC	Area under the curve
LR	Likelihood ratio
PPV	Positive predictive value
NPV	Negative predictive value
